# 

**Published:** 1892-04

**Authors:** 


					﻿Editorial.
The Seventh District Dental Society of Ohio.
The next regular meeting of this society, embracing the south-
western portion of the State, will be held at Washington C. H.,
Tuesday, May 17th, beginning at ten o’clock A. m. and continu-
ing through the day.
The committee having this matter in charge are making
special effort for a good meeting, and it is hoped th^re will be a
large attendance. This is a point easily accessible to all the
counties embraced in this society. Let every member use his
best effort to induce every one possible from his own locality to
be present, and let us have an interesting and profitable meet-
ing. It is here suggested that each an-d every one start in time
to be on hand at the opening of the meeting, even if they leave
home the evening before, and let every one go prepared to add
his part to the interest and profit of the occasion, either prepare
a paper or make some brief notes of cases, modes of practice,
etc., that may elicit discussion, and let every one have more or
less of communication for the question box, which has been a
feature of this society, and let every one, so far as possible, bring
any interesting appliance, instrument, material, etc., that may
be worth attention. The dentist very frequently has something
of this kind, which though very valuable to him, he may think
would not be new and interesting to others ; in this, however,
very frequently we are mistaken for in many such cases these
things are new and interesting to a large number. A minute
description of modes of practice is frequently found of great
value. It is hoped that each member and visitor may have
something of this kind to present on this occasion. So, come
one, come all.
The DENTAL I^EQI^TER,
A. Monthly Magazine Devoted to the Interests of tftk
DENTAL PROFESSION.
Terms Reduced to $2.00 Per Year. Single Copies, 25 Cents.
EDITED BY PROF. J. TAFT, D.D.S.
-----AND-------
Published by SAM’L A. CROCKER R CO., Ohio Dental and Surgical Depot.
The volume commences in January and closes in December.
The Register being issued monthly is always fresh, therefore Indispensable
to the practitioner who desires to keep posted up with the new inventions and
improvements which are daily developed. Neither expense nor effort will be
3pared to give value and variety to its contents. Articles will be illustrated with
well executed engravings whenever they will add to the interest or assist to ex-
planation.
Its extensive circnlat’on and frequency of issue make it one of the BEST DEN-
TAL ADVERTISING MEDIUMS in the United States.
All subscriptions must begin with January or July.
Local or special notices, five lines or less, will be inserted for $3 each insertion ;
aver five lines, 50 ets. per line.
All advertising bills payable in advance, or at furthest, after the issue of the
first number containing the advertisement. Ail transient advertisements to be
?aid for in advince.
««-CONTRIBUTluNS SOLICITED.
Exclusively Editorial Communications should be addressed to the Editor.
The latest number of the Register may be ordered with or without other goods
)* any time, and all letters should be addressed to
aAM’L A. CROCKER & CO , Ohio Dental and Surqical Depot, Cincinnati, 0.
				

